# Antimicrobial prescription at the time of discharge from a tertiary-care hospital in India: A potential target for reducing use at the community level

**DOI:** 10.1017/ash.2022.308

**Published:** 2022-12-09

**Authors:** Stuti Gupta, Jacinta Gunjiyal, Sharin Varma, Rajesh Malhotra, Sharad Shrivastav, Rasna Parveen, Purva Mathur

**Affiliations:** 1 Department of Microbiology, Jai Prakash Narayan Apex Trauma Centre, All India Institute of Medical Sciences, New Delhi, India; 2 Jai Prakash Narayan Apex Trauma Centre, All India Institute of Medical Sciences, New Delhi, India; 3 Department of Orthopaedics, All India Institute of Medical Sciences New Delhi, India

## Abstract

We evaluated the appropriateness of antibiotic prescriptions at discharge from a tertiary-care hospital in India. Of the 790 adult patients included, 84.4% received antibiotics. Microbiological specimens were taken from 67.3% of these patients, and pathogens were identified in 28.8% of cases. Overuse of antimicrobials at hospital discharge should be curtailed.

Antimicrobial resistance (AMR) is an increasingly serious threat to public health globally, with adverse clinical and economic outcomes. A major contributing factor to the rise in AMR includes overuse and/or misuse of antimicrobial agents in hospitals. Approximately half of the consumed antibiotic is eliminated unchanged from the body via feces or urine, which leads to antibiotics seeping into the soil and water. With nearly 35% of the population of India being exposed to feces-contaminated drinking water, this exposure significantly contributes to the development of AMR.^1^

Antibiotic prescribing at discharge from the hospital is common and leads to community transmission of AMR. The Indian Council of Medical Research (ICMR) launched the AMR Surveillance and Research Network in 2013 to facilitate the development of a stewardship program for India.^2^ The All-India Institute of Medical Sciences (AIIMS), New Delhi, is collaborating with the ICMR to decrease hospital-acquired infection (HAI) rates and to build AMS programs across India.^3^ A recently study by Vaughn et al^4^ has provided a framework for antimicrobial stewardship at hospital discharge.

In this study, we evaluated the appropriateness of antibiotic prescriptions at hospital discharge, and we sought to understand how unnecessary prescribing of antibiotics at hospital discharge leads to their misuse at the community level.

## Methods

The study was conducted at a level 1 trauma center of an AIIMS hospital after approval by the institutional ethics committee (no. IEC/89/1/2020). Trauma victims are an otherwise healthy, usually middle-aged population with few underlying diseases. This group is primarily composed of an antibiotic-naïve patients who develop HAIs and AMR due to hospital-related factors.

Data were collected from January to July 2018 to assess the appropriateness of prescriptions concerning the antimicrobial choice, dose, route, frequency, and duration based on standard guidelines and its correlation with microbiology culture practices. Data were analyzed using Stata version 11.1 software (StataCorp, College Station, TX), and results are expressed as mean value and standard deviation (SD) or median and interquartile range (IQR). In most cases, descriptive statistics were used.

## Results and discussion

In total, 790 adult prescriptions were included. The average age of patients in the study was 35.27 years (range, 18–90). The median length of stay was 9 days (range, 1–95). Minor surgical interventions were performed in 93 patients (11.7%) and 448 patients (56.7%) underwent major surgical interventions: orthopedic surgeries (n = 238); abdominal surgery (n = 40); spinal surgery (n = 70); thoracic surgery (n = 8); and polytrauma or grafting surgeries (n = 33). Among these patients, 241 (30.5%) underwent conservative management.

In total, 667 prescriptions (84.4%) were for antibiotics. Among the patients who received them, appropriate microbiological specimens were obtained in 523 cases (67.3%), and a specific pathogen was identified in 214 cases (28.80%). The most commonly identified organisms were *Acinetobacter baumannii* (n = 44, 8.3%), *Pseudomonas aeruginosa* (30, 5.6%), *Escherichia coli* (n = 27, 5.08%), *Klebsiella pneumoniae* (n = 16, 3%), and *Staphylococcus aureus* (n = 26, 4.9%). Other organisms, such as *Citrobacter* spp, *Proteus* spp, *Providentia* spp, *Enterococcus* spp, and *Streptococcal* spp, were also identified. The antimicrobial susceptibility profiles of the most commonly isolated organisms are provided in Table 1. Moreover, 162 patients were diagnosed with infections during their hospitalization. Among them, 72 (10.7%) developed skin and soft-tissue infections, 45 (6.16%) developed respiratory infections, 28 (4.08%) developed bloodstream infections, and 17 (2.41%) developed urinary tract infections. For the patients who received the remaining 123 discharge prescriptions (32.7%), microbiological cultures were not sent to guide the antibiotic therapy. Directed therapy based on susceptibility testing was provided in 74 cases (18%), and 82 patients on antibiotics (12.3%) were culture negative at any time during their hospital stay.

**Table 1.** Antimicrobial Susceptibility Profiles of the Most Commonly Isolated Organisms

Agent	Gram-Negative Bacteria Isolates, No. (%) (N = 168)								
	Enterobacteriaceae			Non-Enterobacteriaceae			Gram-Positive Bacteria Isolates, No. (%) (N = 37)		
	*E. coli*	*K. pneumoniae*	Other Enterobacteriaceae^a^	*Acinetobacter* spp	*Pseudomonas* spp	*Stenotrophomonas maltophilia*	Agent	*Staphylococcus* spp	*Enterococcus* spp
		35 (17.07)	27 (13.17)	9 (5.36)	62 (37.0)	33 (19.7)	3 (1.46)	33 (16.09)	4 (1.95)
Amikacin	23/35 (65.71)	6/27 (22.22)	4/9 (44.4)	6/62 (9.7)	17/33 (51.5)	…	Amoxicillin-clavulanic acid	18/33 (54.5)	0/4 (0)
Cefepime	7/35 (20)	3/27 (11.11)	5/9 (55.6)	1/62 (1.6)	15/33 (45.5)	0/3(0)	Ampicillin	2/33 (6.1)	0/4 (0)
Cefoperazone-sulbactam	20/34 (58.82)	7/27 (25.93)	6/9 (66.7)	6/62 (9.7)	15/33 (45.5)	…	Ampicillin-sulbactam	23/33 (69.7)	0/4 (0)
Cefotaxime	3/23 (13.04)	1/15 (6.67)	2/9 (22.2)	0/62 (0)	7/33 (21.2)		Cefoxitin	13/33 (39.4)	0/4 (0)
Ceftazidime	4/35 (11.43)	2/27 (7.41)	4/9 (44.4)	1/62 (1.6)	13/33 (39.4)	…	Ciprofloxacin	5/33 (15.2)	1/4 (25)
Ceftriaxone	1/29 (3.45)	1/24 (4.17)	3/9 (33.3)	2/62 (3.2)^a^	3/33 (9.1)	…	Clindamycin	22/33 (66.7)	0/4 (0)
Ceftriaxone-sulbactam	19/34 (55.88)	9/27 (33.33)	7/9 (77.8)	6/62 (9.7)	12/33 (36.4)	…	Sulphamethoxazole	13/33 (39.4)	0/4 (0)
Ciprofloxacin	2/35 (5.71)	5/27 (18.52)	5/9 (55.6)	2/62 (3.2)	12/33 (36.4)	…	Erythromycin	17/33 (51.5)	0/4 (0)
Colistin	29/29 (100)	24/27 (88.89)	5/9 (55.6)	60/62 (96.8)	28/33 (84.8)	…	Gentamicin	22/33 (66.7)	1/4 (25)
Gentamicin	16/34 (47.06)	4/27 (14.81)	1/9 (11.1)	5/62 (8.1)	14/33 (42.4)	…	Levofloxacin	10/33 (30.3)	1/4 (25)
Imipenem	30/35 (85.71)	16/26 (61.54)	9/9 (100)	3/62 (4.8)	23/33 (69.7)	…	Linezolid	33/33 (100)	3/4 (75)
Levofloxacin	7/33 (21.21)	6/27 (22.22)	3/9 (33.3)	9/62 (14.5)	12/33 (36.4)	…	Oxacillin	17/33 (51.5)	0/4 (0)
Meropenem	29/35 (82.86)	14/26 (53.85)	7/9 (77.8)	2/62 (3.2)	20/33 (60.6)	0/3 (0)	Penicillin G	1/33 (3)	0/4 (0)
Piperacillin	1/35 (2.86)	2/27 (7.41)	6/9 (66.7)	1/62 (1.6)	13/33 (39.4)	3/3 (100)	Teicoplanin	33/33 (100)	2/4 (50)
Piperacillin-tazobactam	18/33 (54.55)	7/26 (26.92)	6/9 (66.7)	2/62 (3.2)	19/33 (57.6)	1/3 (33.33)	Tetracycline	28/33 (84.8)	1/4 (25)
Tigecycline	34/34 (100)	19/21 (90.48)	8/9 (88.9)	44/62 (71)	10/33 (30.3)	…	Vancomycin	33/33 (100)	2/4 (50)
Trimethoprim-sulphamethoxazole	22/34 (64.71)	9/25 (36)	5/9 (55.6)	0/62 (0)	0/33 (0)	…			

a Other entrobacteriaceae like citrobacter specise, serratia specise, salmonella specise and proteus specise.

**Fig. 1.**
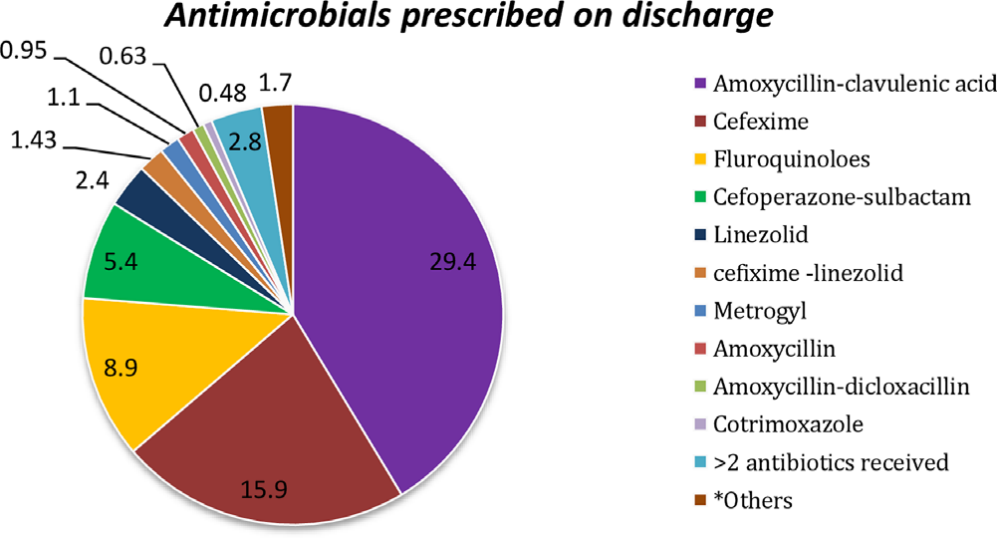
Antimicrobials prescribed on discharge. *Miscellaneous antimicrobials such as clindamycin, metronidazole, azithromycin, doxycycline, netilmicin, rifampicin, amikacin, and ceftriaxone-sulbactam.

Commonly prescribed antibiotics are shown in Figure 1. Overall, 74 prescriptions at discharge (18%) did not mention the exact duration of antibiotics. Also, 29 prescriptions (4.6%) exceeded the recommended duration of antibiotics prescribed. In 43 prescriptions (6.8%), the antibiotic prescribed at discharge was not targeted to organisms isolated in culture. In total, antibiotics with broad gram-negative activity (ie, a fluoroquinolone or amoxicillin-clavulanic acid) were prescribed in 241 cases (38.3%).

Most of the antibiotics were prescribed in the orthopedics department (28%), followed by trauma surgery and critical care units (18.4%), neurosurgery department (13%), and general surgery department (6.6%). However, the rates of culture ordering in these departments were 7%, 9%, 8.3%, and 3% respectively. Most of the restricted antibiotics and combinations of >2 antibiotics were used in the orthopedic department. The median total antibiotic duration was 7 days (IQR, 3–14).

Controlling antibiotic prescription at discharge is increasingly recognized as a potential tool for AMS. Overall, >80% of the patients were discharged with broad-spectrum antibiotics, but culture positivity was only 28% in our study. This overprescribing may be due to fear of developing MDR infections because most patients (66%) had undergone a major surgery. Prescriber behavioral factors, such as fear of treatment failure or readmission, as well as habit may also contribute. Doctors may not account for the intravenous and oral antimicrobial therapy already received by the patient during the hospital stay and thus may simply prescribe a standard 5- or 7-day course of antibiotics upon discharge. AMS programs and repeated training will strengthen the confidence of surgeons in limiting antimicrobial prescriptions.

The overuse of antibiotics is associated with increasing antimicrobial resistance and increased risk of *C. difficile* infection, as well as unnecessary healthcare costs.^5^

Our results contribute to understanding the prescribing pattern of antibiotics at hospital transition and the common errors present at this time, so well-structured strategies can be applied to reduce the overuse of antibiotics. Because most patients in our hospital are trauma patients and their chief complaints are not related to infection, the appropriateness of discharge prescription needs to be further prioritized as an area for AMS interventions.

According to the *Scoping Report on Antimicrobial Resistance in India* (2017), under the aegis of the government of India, among the gram-negative bacteria, >70% of isolates of *E. coli*, *K. pneumoniae*, and *A. baumannii* and nearly half of all *P. aeruginosa* were resistant to fluoroquinolones and third-generation cephalosporins drugs.^6^ These important findings suggest that follow-up cultures and adherence to antibiotic policy regarding the recommendations of antibiotics at discharge should be followed in each hospital. The use of fluoroquinolones in hospitalized patients and upon discharge needs to be monitored and assessed for appropriateness. Few studies have highlighted the impact of fluoroquinolone restriction on the overall reduction of its use and CDI rates.^7,8^ The treatment guidelines issued by the ICMR, which are based on its AMR surveillance data, should be followed when prescribing antibiotics.

Because antimicrobials are so commonly prescribed upon hospital discharge, it is important to identify the patients, indications, and antimicrobials at highest risk of harm from inappropriate therapy. Novel interventions are also needed to reduce the overuse of the antibiotics. Published studies have indicated that improved prescribing can be achieved in many ways: education, involvement of AMS team, electronic prescribing and flagging, generation of alert, culture-based follow-up at discharge, follow-up of patients with inconclusive or pending culture reports, and more.

The findings of this study, along with the paucity of previous studies in India that have addressed this topic, suggest that oral antibiotic prescribing at the transition from inpatient to outpatient care is an important and underrecognized opportunity to reduce the overuse of antibiotics.

## References

[1] Bain R , Cronk R , Hossain R , et al. Global assessment of exposure to faecal contamination through drinking water based on a systematic review. Trop Med Int Health 2014;19:917–927. 24811893 10.1111/tmi.12334 PMC4255778

[2] Walia K , Ohri VC , Mathai D , Antimicrobial Stewardship Programme of ICMR. Antimicrobial stewardship programme (AMSP) practices in India. Indian J Med Res 2015;142:130–138. 26354210 10.4103/0971-5916.164228 PMC4613434

[3] Purva M , Randeep G , Rajesh M , et al. Assessment of core capacities for antimicrobial stewardship practices in Indian hospitals: report from a multicentric initiative of global health security agenda. Indian J Med Microbiol 2019;37:309–317. 32003327 10.4103/ijmm.IJMM_19_445

[4] Vaughn VM , Hersh AL , Spivak ES. Antibiotic overuse and stewardship at hospital discharge: the reducing overuse of antibiotics at discharge home framework. Clin Infect Dis 2022;74:1696–1702. 34554249 10.1093/cid/ciab842 PMC9070833

[5] Yogo N , Haas MK , Knepper BC , et al. Antibiotic prescribing at the transition from hospitalization to discharge: a target for antibiotic stewardship. Infect Control Hosp Epidemiol 2015;36:474–478. 25782905 10.1017/ice.2014.85 PMC4841620

[6] Gandra S , Joshi J , Trett A , Lamkang A , Laxminarayan R. Scoping Report on Antimicrobial Resistance in India. Washington, DC: Center for Disease Dynamics, Economics & Policy; 2017.

[7] Shea KM , Hobbs ALV , Jaso TC , et al. Effect of a healthcare system respiratory fluoroquinolone restriction program to alter utilization and impact rates of *Clostridium difficile* infection. Antimicrob Agents Chemother 2017;61:e00125–17. 28348151 10.1128/AAC.00125-17 PMC5444144

[8] Sarma JB , Marshall B , Cleeve V , Tate D , Oswald T , Woolfrey S. Effects of fluoroquinolone restriction (from 2007 to 2012) on *Clostridium difficile* infections: interrupted time-series analysis. J Hosp Infect 2015;91:74–80. 26169793 10.1016/j.jhin.2015.05.013

